# Crossroads of Antimicrobial and Diagnostic Stewardship: Assessing Risks to Develop Clinical Decision Support to Combat Multidrug-Resistant *Pseudomonas*

**DOI:** 10.1093/ofid/ofad512

**Published:** 2023-10-12

**Authors:** Iris Zou, Daniel Abate, Michelle Newman, Emily L Heil, Surbhi Leekha, Kimberly C Claeys

**Affiliations:** Department of Nursing, University of Maryland Medical Center, Baltimore, Maryland, USA; Department of Pharmacy, Baltimore Washington Medical Center, Baltimore, Maryland, USA; Department of Epidemiology and Public Health, University of Maryland School of Medicine, Baltimore, Maryland, USA; Department of Practice and Health Outcomes Research, University of Maryland School of Pharmacy, Baltimore, Maryland, USA; Department of Epidemiology and Public Health, University of Maryland School of Medicine, Baltimore, Maryland, USA; Department of Practice and Health Outcomes Research, University of Maryland School of Pharmacy, Baltimore, Maryland, USA

**Keywords:** antibiotic resistance, antimicrobial stewardship, diagnostic stewardship, *Pseudomonas aeruginosa*

## Abstract

**Background:**

Early detection of multidrug-resistant *Pseudomonas aeruginosa* (MDRP) remains challenging. Existing risk prediction tools are difficult to translate to bedside application. The goal of this study was to develop a simple electronic medical record (EMR)–integrated tool for prediction of MDRP infection.

**Methods:**

This was a mixed-methods study. We conducted a split-sample cohort study of adult critical care patients with *P aeruginosa* infections. Two previously published tools were validated using c-statistic. A subset of variables based on strength of association and ease of EMR extraction was selected for further evaluation. A simplified tool was developed using multivariable logistic regression. Both c-statistic and theoretical trade-off of over- versus underprescribing of broad-spectrum MDRP therapy were assessed in the validation cohort. A qualitative survey of frontline clinicians assessed understanding of risks for MDRP and potential usability of an EMR-integrated tool to predict MDRP.

**Results:**

The 2 previous risk prediction tools demonstrated similar accuracy in the derivation cohort (c-statistic of 0.76 [95% confidence interval {CI}, .69–.83] and 0.73 [95% CI, .66–.8]). A simplified tool based on 4 variables demonstrated reasonable accuracy (c-statistic of 0.71 [95% CI, .57–.85]) without significant overprescribing in the validation cohort. The risk factors were prior MDRP infection, ≥4 antibiotics prior to culture, infection >3 days after admission, and dialysis. Fourteen clinicians completed the survey. An alert providing context regarding individual patient risk factors for MDRP was preferred.

**Conclusions:**

These results can be used to develop a local EMR-integrated tool to improve timeliness of effective therapy in those at risk of MDRP infections.

*Pseudomonas aeruginosa* is a difficult-to-treat pathogen due to its ability to develop antibiotic resistance through multiple mechanisms, including decreased expression of outer membrane porins, upregulation of efflux pumps, and β-lactamase production [1–4]. Infections caused by *P aeruginosa*, particularly bloodstream and lower respiratory tract infections, are associated with worse patient outcomes when multidrug-resistant *P aeruginosa* (MDRP) is isolated [5–7]. In data from the Centers for Disease Control and Prevention (CDC) and JMI Laboratories, among 2422 *P aeruginosa* isolates from blood and respiratory cultures, nonsusceptibility to piperacillin-tazobactam or meropenem approached 20% [8]. When selecting for critically ill patients, these rates of resistance increase substantially. Not surprisingly, the CDC has listed MDRP as a serious public health threat [9].

Delayed effective therapy for infections caused by *P aeruginosa* is associated with significantly increased odds of 30-day mortality [10]. Start of effective treatment for MDRP, such as ceftolozane-tazobactam, has been found to be delayed up to a median of 9 days [11]. While detection of genetic resistance determinants, particularly β-lactamases, using rapid diagnostic tests can assist in earlier optimization of antibiotic therapy, their use is limited in MDRP detection due to multifactorial mechanisms of resistance beyond β-lactamase production [12]. Therefore, risk scoring tools based on clinical characteristics can be important for prediction of MDRP to optimize antibiotic use earlier and improve patient outcomes [13–17]. However, available tools need to be locally validated to determine utility and impact in a given clinical setting [18, 19]. Furthermore, current tools used to predict MDRP have many input factors, limiting their bedside usability and clinician acceptability [13, 15]. In this study, we created a clinically relevant, site-specific, risk scoring tool using predictive characteristics from our hospital's critically ill patient population with the goal to develop a user-centered tool that can be easily integrated into the electronic medical record (EMR) and existing clinical workflow.

## METHODS

### Study Design

This is a mixed-methods study consisting of 2 parts: (1) a split-sample retrospective cohort study to develop and validate a localized MDRP risk prediction tool and (2) a survey of end-user clinicians to determine understanding of risk of MDRP infection and acceptability of using an EMR-integrated tool to predict the risk of MDRP and guide empiric therapy selection.

### Part 1

#### Study Setting and Population

This single-center, retrospective cohort study took place at the University of Maryland Medical Center, a 757-bed urban tertiary care teaching hospital. The study sample consisted of adult inpatients (≥18 years of age) who received care in an intensive care unit (ICU) and had at least 1 positive clinical (not surveillance) culture for *P aeruginosa* from blood, sputum, or bronchoalveolar lavage (BAL). Patients were excluded if antimicrobial susceptibility data were not available, patients were not diagnosed with a clinical infection, or researchers were unable to obtain variables determined to be associated with risk of MDRP. Clinical diagnosis of infection was determined through medical record review based on treating clinician documentation in the EMR (EPIC, Verona, Wisconsin).

To align with local clinical practices, the institutional classification of MDRP was used in this study. MDRP was defined as *P aeruginosa* that was nonsusceptible to at least 1 agent in 2 of 3 β-lactam antimicrobial classes: antipseudomonal carbapenems, piperacillin-tazobactam, or antipseudomonal cephalosporins. Of note, this is different than the more strict Infectious Diseases Society of America definition of *P aeruginosa* with difficult-to-treat resistance, which is nonsusceptible to piperacillin-tazobactam, ceftazidime, cefepime, aztreonam, meropenem, imipenem-cilastatin, ciprofloxacin, and levofloxacin [4]. All cultures were processed on-site using the VITEK MS and VITEK 2 automated susceptibility systems (bioMérieux, Durham, North Carolina). Phenotypic nonsusceptibility was based on Clinical and Laboratory Standards Institute breakpoints for *P aeruginosa* [20].

Two cohorts of patients were included, a derivation cohort and a validation cohort. The derivation cohort of patients was admitted from July 2017 to May 2019 and was used to locally validate previously published risk scoring tools developed by Tartof et al [13] and Lodise et al [14]. This cohort was also used to assess individual risk factors from these previously published tools to develop a simplified risk scoring tool for isolation of MDRP in our local patient population. A validation cohort of patients admitted from July 2019 to April 2021 was then collected to test the accuracy of the newly developed simplified risk tools and determine clinical trade-off of over- versus underprescribing broad-spectrum MDRP antimicrobials based on specific risk score cutoffs. Of note, during the validation study period, those with COVID-19 infection identified during hospitalization were excluded from the analysis. A comparison of previously published risk scores and risk factors evaluated is available in Table 1. For this study, all data on risk factors were collected through manual review of the EMR. Definitions for risk factor variables are provided in the Supplementary Appendix.

**Table 1.** Comparison of Previously Published Risk Scoring Tools

Characteristic	Risk Scoring Tool 1 (Lodise et al [14])	Risk Scoring Tool 2 (Tartof et al [13])	
Patient population	Premier Hospital Database (500 acute-care hospitals across the US)	Kaiser Permanente Southern California (14 hospitals)	
Study sample	124 068 patients	Split-sample, 11 502 infections (5834 and 5668)	
Definition of MDRP	Nonsusceptible to at least 3 antipseudomonal agents, including penicillins, cephalosporins, monobactams, carbapenems, aminoglycosides, or fluoroquinolones	(1) CR: Nonsusceptible to meropenem or imipenem	(2) Extensive β-lactam resistance: Nonsusceptible to meropenem or imipenem, ceftazidime, and piperacillin-tazobactam
Incidence of MDRP	3.91%	20.2%	8.9%
Risk score	Site of infection Admitted >72 h ICU at culture collection Prevalence of MDRP Patient age Prior admission in last 6 mo Prior infection in last 3 mo Prior number of antibiotics in current admission Admission source (ie, transfer) Dialysis Diabetes with complications Diabetes without complications Peripheral vascular disease Paraplegia and hemiplegia Chronic pulmonary disease Myocardial infarction Cancer Cerebrovascular disease Congestive heart failure Mild liver disease	History of PsA infection (30 d) History CR PsA infection (30 d) No PsA infection (30 d) Carbapenem use (30 d) Cephalosporins (30 d) Quinolones (30 d) SNF transfer Tracheostomy Previous hospitalization (6 mo)	History of PsA infection (30 d) History of CR PsA infection (30 d) Carbapenem use (30 d) SNF transfer Tracheostomy CVC, PICC, or port Previous hospitalization (6 mo)
Prediction output	Percentage risk of infection, 0% to 100%	Cumulative score from −3 to 20 points	Cumulative score from 0 to 19 points
Performance (training data)	AUC ROC = 0.94	AUC ROC = 0.82 (95% CI, .80–.83)	AUC ROC = 0.84 (95% CI, .82–.86)

Abbreviations: AUC ROC, area under the receiver operating characteristic curve; CI, confidence interval; CR, carbapenem resistant; CVC, central venous catheter; ICU, intensive care unit; MDRP, multidrug-resistant *Pseudomonas aeruginosa*; PICC, peripherally inserted central catheter; PsA, *Pseudomonas aeruginosa*; SNF, skilled nursing facility; US, United States.

#### Patient Consent Statement

This study was approved by the University of Maryland Baltimore Institutional Review Board and received a waiver of informed consent due to the retrospective nature of the study. This study was conducted in compliance with the Health Insurance Portability and Accountability Act.

#### Study Outcomes

The first objective was to determine the accuracy of previously published risk scoring tools in predicting presence of MDRP among critically ill patients with clinical cultures from the blood or respiratory tract with *P aeruginosa* identified. The second objective was to determine fidelity of each risk factor and develop a new, pragmatic, and locally validated risk tool to predict MDRP infection within our local patient population. To determine the fidelity of risk factors from the previously published risk scoring tools, we assessed each parameter's individual association with MDRP isolation in the derivation cohort. To develop a new risk scoring tool, we chose those parameters (1) that were most strongly associated with MDRP isolation and (2) that could be extracted from the EMR, to include in a new risk scoring tool that could be tested further in the validation cohort.

#### Statistical Analysis

For bivariate analysis, categorical variables (ie, demographics, clinical history, and comorbid conditions) were compared using Fisher exact test or χ^2^ test as appropriate, and continuous variables (age, length of stay) were compared using Student *t* test or Mann-Whitney *U* test as appropriate. A *P* value of <.05 was considered statistically significant and all analyses were 2-tailed. For individual risk factors, unadjusted odds ratios (ORs) and accompanying 95% confidence intervals (CIs) were calculated. Those with the strongest association with isolation of MDRP in the derivation cohort were considered for inclusion in the risk scoring tools. Multivariable backwards logistic regression was conducted to determine independent predictors of MDRP. To achieve a parsimonious and pragmatic model, we limited the number of variables, with special consideration for those that can be automatically pre-populated from the EMR.

Risk factors identified from the multivariable regression model were used to construct multiple risk scoring tools. The β-coefficients from the regression model were standardized to a weight-proportional integer value to be included in the scoring tools. To assess the overall accuracy of these risk scoring tools, c-statistic was computed, first within the derivation and then validation cohort. To better express clinical utility of the risk scoring tools, a decision analytic approach was taken to determine the appropriate cutoff value to maximize net benefit that would provide optimal balance between overtreatment and undertreatment with respect to prescription of ceftolozane-tazobactam based on predetermined score cutoffs. Multiple combinations of the predetermined risk factors with weighted points were examined in both the derivation and validation cohorts to determine overall accuracy through c-statistic as well as determine a cutoff point to trigger empiric prescribing of newer broad-spectrum MDRP therapy such as ceftolozane-tazobactam.

### Part 2

#### Clinician Recruitment, Data Collection, and Statistical Analysis

The objective of this part of the study was to determine clinician baseline understanding of risk factors for MDRP infection and acceptability EMR-integrated clinical decision support to improve awareness of possible MDRP infection requiring broad empiric antimicrobial therapy. The survey was open from 12 to 28 June 2023, with an introductory recruitment email followed by 3 follow-up emails. We recruited frontline providers involved in initiating or adjusting empiric antibiotic therapy in the ICUs at University of Maryland Medical Center. These end-users included physicians at a variety of training levels (attendings, residents, and fellows) and advanced practice practitioners in critical care and infectious diseases. Participation was voluntary and confidential. A waiver of documentation of informed consent was obtained. Participants who completed the survey received an electronic gift card.

**Figure 1.**
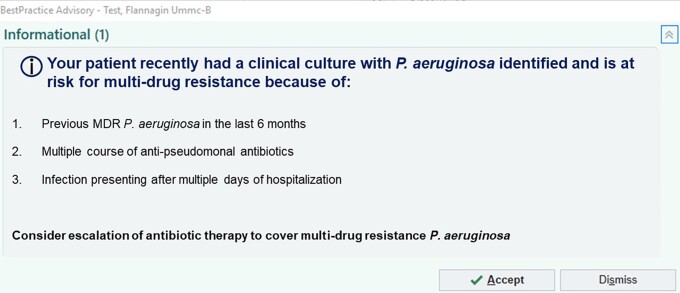
Example of prototype clinical decision support tool. Abbreviation: MDR, multidrug resistant.

The survey was developed using the REDCap survey tool [21]. Survey questions consisted of 18 questions and focused on understanding of risk factors for MDRP and decisions on initiating empiric broad-spectrum therapy in those at risk for MDRP (Supplementary Appendix). Within the survey we presented 3 low-fidelity visual display prototypes of proposed clinical decision support tools to aid in determining those at highest risk for MDRP and asked participants about perceived barriers and facilitators to using these tools to guide empiric therapy decisions (Figure 1). Responses to survey results, including questions on the Likert scale, were summarized using descriptive statistics, including frequencies and percentages.

## RESULTS

### Study Derivation Population

For the derivation cohort, 352 adult inpatients admitted between June 2017 and May 2019 were screened for inclusion; 282 met the inclusion criteria. The mean age was 56.5 (standard deviation [SD], 15.9) years, and 168 (60%) were male. The median time to collection of cultures with *P aeruginosa* isolated from admission was 7 (interquartile range [IQR], 2–18) days. Sputum was the most common specimen source (157 [56%]), followed by BAL (90 [32%]). Among the derivation cohort, 66 (23%) patients had an MDRP based on the study definition. Carbapenem-resistant *P aeruginosa* accounted for the majority of MDRP (41 [62%]) (Table 2).

**Table 2.** Comparison of Clinical Characteristics and Risk Factors Among Patients With and Those Without Multidrug-Resistant *Pseudomonas aeruginosa*

Characteristic	MDRP		
	No (n = 216)	Yes (n = 66)	*P* Value
Patient age group, y			
18–25	13 (6)	3 (4.5)	.735
26–35	19 (8.8)	9 (12.1)	
36–45	13 (6)	3 (4.5)	
46–55	45 (19.4)	8 (12.1)	
56–65	63 (29.2)	23 (34.8)	
≥66	66 (30.6)	21 (31.8)	
Admission source			
Community	118 (54.6)	32 (48.5)	.111
Outside hospital	79 (36.6)	22 (33.3)	
Skilled nursing/long-term care facility	18 (8.3)	10 (15.2)	
Other	1 (0.5)	2 (3)	
Infection <72 h (POA)	77 (35.6)	9 (13.6)	<.001
Infection type			
Bloodstream	25 (11.6)	5 (7.6)	.328
HBAP/VAP	137 (63.4)	50 (75.8)	
Other	20 (9.2)	4 (6.1)	
Unknown	34 (15.7)	7 (10.6)	
Previous hospital admission (6 mo)	77 (35.6)	38 (57.6)	.002
Previous ICU admission (6 mo)	31 (14.4)	23 (34.8)	<.001
Previous PsA infection (30 d)			
Previous MDRP (30 d)	0 (0)	1 (1.5)	NS
Previous MDRP (6 mo)	2 (0.9)	5 (7.6)	.002
Previous MDRGN (6 mo)	8 (3.7)	13 (19.7)	<.001
Previous antibiotics (30 d)	173 (80.1)	62 (93.9)	.008
Unique antibiotics received prior to PsA culture			
0–1	75 (34.7)	3 (4.5)	<.001
2–3	81 (37.5)	19 (28.8)	
≥4	60 (27.8)	44 (66.7)	
Receipt of antipseudomonal agent			
Fluoroquinolone	17 (7.9)	12 (18.2)	<.001
Carbapenem	27 (12.5)	30 (45.5)	
Piperacillin-tazobactam	71 (32.9)	40 (60.6)	
Cefepime	34 (15.7)	26 (39.4)	
Invasive lines			
Receipt of ≥2 antipseudomonal agents	34 (15.7)	34 (51.5)	< .001
Tracheostomy prior to PsA culture	47 (21.8)	30 (45.5)	<.001
Endotracheal tube prior to PsA culture	182 (84.3)	45 (68.2)	.004
Hemodialysis prior to PsA culture	39 (84.3)	32 (48.5)	.064
PICC, CVC, or port prior to PsA	126 (58.3)	46 (67.9)	.098
Comorbidities and past medical history			
Chronic pulmonary disease	58 (26.9)	25 (37.9)	.085
Diabetes with complications^a^	14 (6.5)	11 (16.7)	.011
Diabetes without complications	40 (18.5)	20 (30.3)	.041
Dialysis	22 (10.2)	20 (30.3)	<.001
Cancer	24 (11.1)	7 (10.6)	.909
Myocardial infarction	7 (7.9)	4 (6.1)	.624
Mild liver disease	17 (7.9)	10 (15.2)	.079
Congestive heart failure	29 (13.4)	14 (21.2)	.124
Peripheral vascular disease	10 (4.6)	3 (4.5)	.977
Cerebrovascular disease	46 (21.3)	9 (13.6)	.169
Paraplegia or hemiplegia	3 (1.4)	5 (7.6)	.008

Data are presented as No. (%).

Abbreviations: CVC, central venous catheter; HBAP/VAP, hospital-acquired bacterial pneumonia/ventilator-associated bacterial pneumonia; ICU, intensive care unit; MDRGN, multidrug-resistant gram-negative; MDRP, multidrug-resistant *Pseudomonas aeruginosa*; NS, not significant; PICC, peripherally inserted central catheter; POA, present on admission; PsA, *Pseudomonas aeruginosa*.

^a^Complications include the presence of microvascular (ie, retinopathy, nephropathy, and neuropathy) or macrovascular complications.

In the derivation cohort, the Lodise et al risk scoring tool had a c-statistic of 0.76 (95% CI, .69–.83). The median risk of MDRP in the derivation population was 22%, ranging from 0% to 89%. Among those with MDRP, the median predicted probability was 53% (IQR, 24%–66.5%) compared with 16% (IQR, 4%–35.7%) who had non-MDRP (*P* < .001). Using the carbapenem-resistant *P aeruginosa* risk scoring tool from Tartof et al to predict MDRP, the c-statistic in the same derivation cohort was 0.74 (95% CI, .66–.81). In this cohort, the median score was 2 (range, 0–20). Applying this tool to only those with MDRP defined as carbapenem resistant, the c-statistic was 0.8 (95% CI, .73–.88). Among those with MDRP isolates, the median score was 6 (IQR, 2–9), compared with 1.5 (IQR, 0–4) among those who had non-MDRP (*P* < .001). Using the extensive β-lactam resistance *P aeruginosa* risk scoring tool from the same investigators, the c-statistic was 0.731 (95% CI, .66–.8) and the median score was 4 (range, 0–19). Among those with MDRP isolates, the median score was 6 (IQR, 3–10) compared with 3 (IQR, 0.5–5) among those who had non-MDRP (*P* < .001).

**Figure 2.**
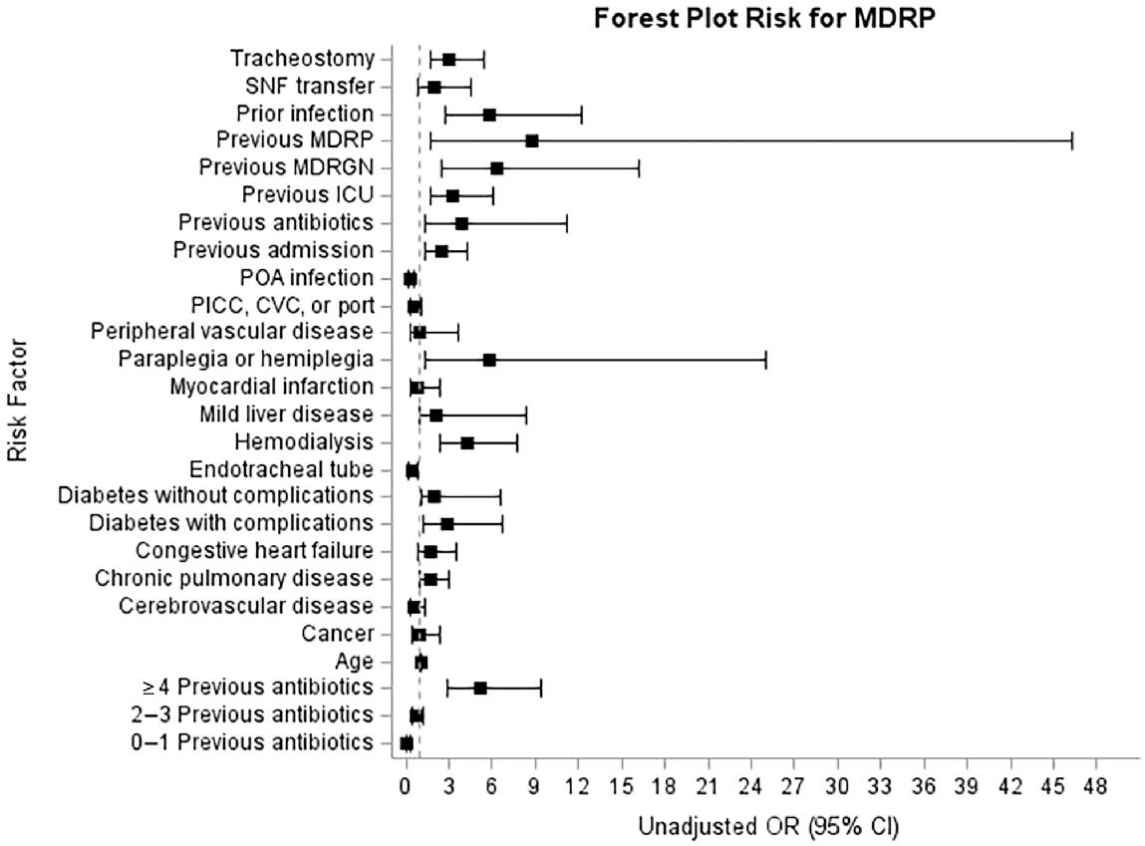
Risk factors associated with multidrug-resistant *Pseudomonas aeruginosa* infection. Abbreviations: CI, confidence interval; CVC, central venous catheter; ICU, intensive care unit; MDRGN, multidrug-resistant gram-negative; MDRP, multidrug-resistant *Pseudomonas aeruginosa*; OR, odds ratio; PICC, peripherally inserted central catheter; POA, present on admission; SNF, skilled nursing facility.

In the logistic regression analysis in our derivation cohort, risk factors with the highest odds of association with MDRP were prior MDRP (OR, 8.7), prior MDR gram-negative (OR, 6.4), paraplegia or hemiplegia (OR, 5.8), prior *P aeruginosa* infection in the last 3 months (OR, 5.8), receiving ≥4 antibiotics before index culture date during hospitalization (OR, 5.2), hemodialysis (OR, 4.3), previous antibiotics in the last 30 days (OR, 3.9), and infection not present on admission (OR, 3.5) (Figure 2). From the evaluated risk factors with highest odds, the following 5 were selected for further evaluation in a series of logistic regression models: (1) prior MDRP infection; (2) ≥4 previous antibiotics before index culture; (3) infection not present on admission; and (4) dialysis. These variables were selected based both on strength of association and ease of automatic extraction from the EMR.

### Study Validation Population

Data for the validation cohort were collected from 75 patients from July 2019 to April 2021. The mean age of these patients was 56.6 (SD, 13) years, and 59 (79%) were male. The median time to index culture with identification of *P aeruginosa* from admission was 11 (IQR, 5–15) days. BAL was the most common specimen source (72 [96%]), followed by sputum (3 [4%]). Among the validation cohort, 16 (21.3%) patients had an MDRP based on the study definition.

Multiple combinations of the predetermined risk factors above with weighted points were tested in 4 separate risk scoring tools to examine overall accuracy by c-statistics as well as potential score cutoff points that would potentially trigger recommendations to change empiric prescribing for broad-spectrum MDRP therapy (Table 3). The score with the highest accuracy (score 2) had a c-statistic of 0.73 (95% CI, .66–.8) for the derivation cohort and 0.71 (95% CI, .57–.85) for the validation cohort. With a score cutoff point of 9 of 17, a score >9 indicated a high risk of MDRP infection. Applied to this validation cohort, the score would not result in excess overprescribing (0%) but would miss 19.2% of MDRP.

**Table 3.** Simplified Risk Scoring Tools Among the Validation and Derivation Cohorts

	Proposed Score A	Proposed Score B	Proposed Score C	Proposed Score D
Risk factor	≥4 Abx Not POA Prior MDRP	≥4 Abx Not POA Prior MDRP Inpatient dialysis	≥4 Abx Prior MDRP Inpatient dialysis	≥4 Abx Not POA Prior MDRGN
Accuracy, c-statistic (95% CI)	V: 0.73 (.66–.8) D: 0.7 (.55–.85)	V: 0.73 (.66–.8) D: 0.71 (.57–.85)	V: 0.72 (.64–.79) D: 0.69 (.53–.84)	V: 0.72 (.65–.78) D: 0.68 (.53–.82)
Score cutoff	6/14 points	9/17 points	9/15 points	6/10 points

Abbreviations: Abx, antibiotics; CI, confidence interval; D, derivation cohort; MDRGN, multidrug-resistant gram-negative; MDRP, multidrug-resistant *Pseudomonas aeruginosa*; POA, present on admission; V, validation cohort.

### Frontline Clinician Survey

**Figure 3.**
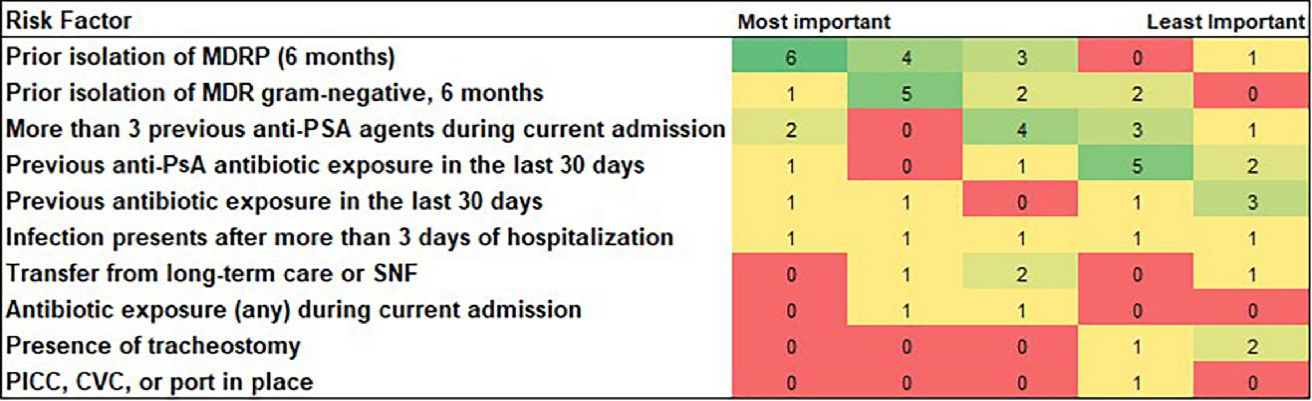
Clinical importance of risk factors for multidrug-resistant *Pseudomonas aeruginosa* when prescribing empiric antibiotic therapy. Abbreviations: CVC, central venous catheter; MDRP, multidrug-resistant *Pseudomonas aeruginosa*; PICC, peripherally inserted central catheter; PsA, *Pseudomonas aeruginosa*; SNF, skilled nursing facility.

Of a total of 77 providers invited to participate, there were 14 survey respondents, for a response rate of 18%. Many respondents reported practicing in the surgical ICU (11 [78.6%]), followed by neurosurgical ICU (6 [42.8%]) and medical ICU (5 [35.7%]). Years in practice were evenly distributed between 1–3 years (4 [28.6%]), 3–5 years (6 [42.9%]), and 5–10 years (4 [28.6%]). All respondents were either ICU attendings (5 [35.7%]) or fellows (9 [64.3%]). Major drivers for prescribing broad empiric therapy targeting MDRP, such as ceftolozane-tazobactam, include progression/worsening of illness (11 [78.6%]), septic shock (8 [57.1%]), and past MDR gram-negative noted by Infection Prevention in the EMR and/or past surveillance cultures (8 [57.1%]). Respondents were also asked to rank the most important factors for determining if a patient is at risk of MDRP (Figure 3). Major decision aids for prescribing broad empiric therapy targeting MDRP included refence to local guidelines (10 [71%]), local antibiograms (11 [79%]), infectious diseases consult recommendations (9 [64%]), and EMR-based order sets (4 [29%]).

Three different prototypes of clinical decision support within the EMR were presented to respondents: a banner report, a best practice alert (BPA), and a preliminary culture report nudge. The majority of respondents agreed or strongly that a BPA that alerted to the risk of MDRP would aid in clinical decision making (10 [71.4%]). Respondents noted that providing the patient-specific risk factors that prompted the alert makes this tool more useful, but also noted that general alert fatigue may limit interaction with the tool and to avoid excessive need to “click through” multiple screens (eg, avoid overly complex clinical decision support tools). Tying the alert to specific antimicrobials that may be ordered empirically for treatment of sepsis, such as piperacillin-tazobactam, was seen as a viable way to spread the message of potential need to broaden therapy. Respondents neither agreed or disagreed on the report nudge being useful to decision (6 [42%]), noting that the report cannot detail why the patient is at risk (not enough clinical context) and may confuse individuals reading the preliminary results without susceptibility data. A banner alerting to the risk of MDRP was seen as least useful to guide empiric therapy decisions. The need for providing education on any tool prior to implementation was noted by many respondents.

## DISCUSSION

The goal of this study was to develop a contextually relevant, simplified, risk scoring tool that could help predict infection with MDRP and improve empiric antimicrobial therapy decisions. The proposed tool, built using risk factors from previously published risk scoring tools and validated with local data, consists of 4 risk factors that could be automatically extracted from the EMR (≥4 previous antibiotics during admission, infection was not present on admission, prior MDRP, and inpatient renal replacement therapy). This tool demonstrated similar accuracy as more complex risk scores and would not result in excessive overprescribing of broad empiric therapy. Providers agreed that an alert notifying why their patient was at risk for MDRP, coupled with education on appropriate prescribing, would be most effective while limiting impact on workflow.

Use of risk scoring tools can be challenging as they are often developed as an academic exercise with limited ability to be used beside. They also require local validation prior to implementation. The first tool was developed using split-sample validation of a dataset provided by Kaiser Permanente Southern California that contained >11 000 patients with *P aeruginosa* infections [13]. The authors noted that they were able to create parsimonious risk scoring tools; however, external validation and assessment for meaningful cutoff values was still needed before successful integration into clinical practice. The second tool, developed from >120 000 adult patients using Premier data, contains multiple predictive models for MDR gram-negatives, including a model to determine the risk of MDRP [14]. This tool was internally validated and noted to have been implemented within several healthcare facilities to guide early appropriate therapy. While increasing its accuracy, the 19 metrics that must be entered into the tool can be time-consuming and interrupt workflow and may serve as a hindrance to practical widespread implementation. Additionally, the tool is contained within a separate Excel file, which may decrease provider uptake compared to a tool that is integrated into an existing EMR.

To assist in determining potential uptake of an EMR-integrated risk scoring tool, we surveyed frontline clinicians to determine potential barriers to use. We found from survey responses that frontline clinicians can identify the most important risk factors for MDRP when provided with a defined list. However, challenges may arise when trying to practically apply these risk factors to antibiotic therapy decisions at the bedside, especially when patient-related risks may not be easy to readily determine. Those interviewed identified that a simple informational nudge would be most effective in conveying the appropriate contextual factors needed to make empiric therapy decisions while limiting interruptions to workflow. Nudges are frequently used in clinical microbiology to guide decision making and are often incorporated into microbiology result reporting through informational comments or selective reporting of antimicrobial susceptibilities [22]. Electronic alerts nudging prescribers to reconsider therapy decisions earlier in the diagnostic process have also been studied [23]. By reinforcing what providers already understand about risk for MDRP, inserting a simple educational nudge can positively influence empiric therapy decisions. A notable limitation of the current survey is the small sample size of respondents. Additionally, the survey was focused on prescribers in our ICUs, who were most likely to interact with such a tool, so the range of opinions is limited.

To overcome the challenge of clinical accessibility, we simplified the predictive model to several metrics that are easily extractable from the EMR without sacrificing the accuracy of predicting MDRP in our institution's population. However, as with any risk assessment, the accuracy in predicting MDRP is limited in generalizability and the tool was imperfect in predicting all MDRP. Therefore, it should be used in conjunction with an assessment of the patient's overall clinical presentation. Other limitations include the single-center focus of our study and our inability to be comprehensive in evaluation of past health and antibiotic exposure beyond what was reported in the EMR. Antibiotic resistance rates change over time, so the retrospective nature of our study is also potentially confounded by time. The methods used in this study can be applied by other sites, but other institutions should evaluate the ORs of certain risk factors in their patient populations since institutions differ in their case-mix index and rate of multidrug resistance.

## CONCLUSIONS

Previous risk scoring tools included numerous metrics, which may make them difficult to use in the clinical setting. There is a need for simple, locally validated risk scoring tools to quickly predict MDRP. Our study demonstrated that a clinically relevant simplified risk score can be developed by using a retrospective cohort to identify relevant institution-specific risk factors. Next steps for clinical accessibility include integration as a clinical support tool in the EMR to automate detection of risk factors. Future research is needed to evaluate the effectiveness of implementing such a tool into the clinical setting and whether the tool will positively impact prescribing behaviors. Methods outlined in this study can be reproduced to create a clinically relevant site-specific tool at other institutions to improve management of MDRP.

## Supplementary Data

Supplementary materials are available at *Open Forum Infectious Diseases* online. Consisting of data provided by the authors to benefit the reader, the posted materials are not copyedited and are the sole responsibility of the authors, so questions or comments should be addressed to the corresponding author.

## Supplementary Material

ofad512_Supplementary_Data

## Notes

***Financial support.*** This research was supported by funding from Merck Sharp & Dohme LLC, a subsidiary of Merck & Co, Inc, through the Merck Investigator Studies Program. Payments were made to institution with study author K. C. C. as principal investigator.
