# Supplementary material for: Crossroads of Antimicrobial and Diagnostic Stewardship: Assessing Risks to Develop Clinical Decision Support to Combat Multidrug-Resistant Pseudomonas
Source: Open Forum Infect Dis. 2023 Oct 12;10(10):ofad512. doi: 10.1093/ofid/ofad512 (PMC10603593; doi:10.1093/ofid/ofad512)
Supplement: ofad512_Supplementary_Data [file ofad512_supplementary_data.zip › Survey_ClinicalDecisionSupport.pdf]

Survey

Please complete the survey below.

Thank you!

The purpose of this study is to help improve the safety and value of care for patients at risk for multi-drug resistant (MDR) P. aeruginosa.

We are working on incorporating risk factors for MDR P. aeruginosa into the electronic medical record (EMR) to improve empiric prescribing of effective antibiotics. We will be asking for your feedback and input on some new strategies and tools to alert clinicians of patients at high risk for MDR P. aeruginosa.

There are no right or wrong answers. The information you provide will be used to further assess and refine these tools as well as mitigate barriers to use by clinicians. Results of the survey will be confidential.

PART 1

We would like to begin by better understanding current processes for respiratory culture ordering and antibiotic initiation.

After blood and/or respiratory cultures have been collected, how are decisions made regarding empiric antibiotic therapy? Are any decision aids used in this process?

Check all that apply

- ☐ Local clinical guidelines
- ☐ Local antibiograms
- ☐ EMR-based order-sets
- ☐ Best-practice alerts
- ☐ ID consult
- ☐ Other

Describe if 'other'

____

Are the below criteria appropriate to use to determine if a patient is at risk for MDR P. aeruginosa ?
PLEASE RANK YOUR TOP 5 CHOICES

	1	2	3	4	5
1. Previous antibiotic exposure in the last 30 days (any)	○	○	○	○	○
2. Previous anti-PSA antibiotic exposure in the last 30 days	○	○	○	○	○
3. More than 3 previous anti-PSA agents during current admission	○	○	○	○	○
4. Antibiotic exposure (any) during current admission	○	○	○	○	○
5. Transfer from long-term care or SNF	○	○	○	○	○
6. Infection presents after more than 3 days of hospitalization	○	○	○	○	○
7. PICC, CVC, or port in place	○	○	○	○	○

8. On renal replacement therapy	○	○	○	○	○
9. Presence of tracheostomy	○	○	○	○	○
10. Advanced age	○	○	○	○	○
11. Prior isolation of MDR Gram-negative (clinical) within the last 6 months	○	○	○	○	○
12. Prior isolation of MDR P. aeruginosa (clinical or surveillance) within the last 6 months	○	○	○	○	○

When do you consider initiating EMPIRIC broad spectrum antibiotic therapy with activity against MDR P. aeruginosa such as ceftolozane/tazobactam?

Check all that apply

- ☐ Patient acuity (i.e., septic shock, ventilation requirements)
- ☐ Progression of illness on antibiotic therapy
- ☐ Past P. aeruginosa infection in the last 3 to 6 months
- ☐ Past MDR Gram-negative noted by Infection Prevention and/or past survellance cultures
- ☐ ID consult recommends empiric initiation
- ☐ Other

Describe if 'other'

____

Does your AMS program, to the best of your awareness, have interventions geared towards the appropriate treatment of MDR P. aeruginosa?

- ○ Yes
- ○ No
- ○ Unsure

PART 2
We would like to get your feedback on strategies and tools to help clinicians determine if the risk of MDR P. aeruginosa is sufficient to warrant empiric use of broad-spectrum antibiotic agents such as ceftolozane/tazobactam.

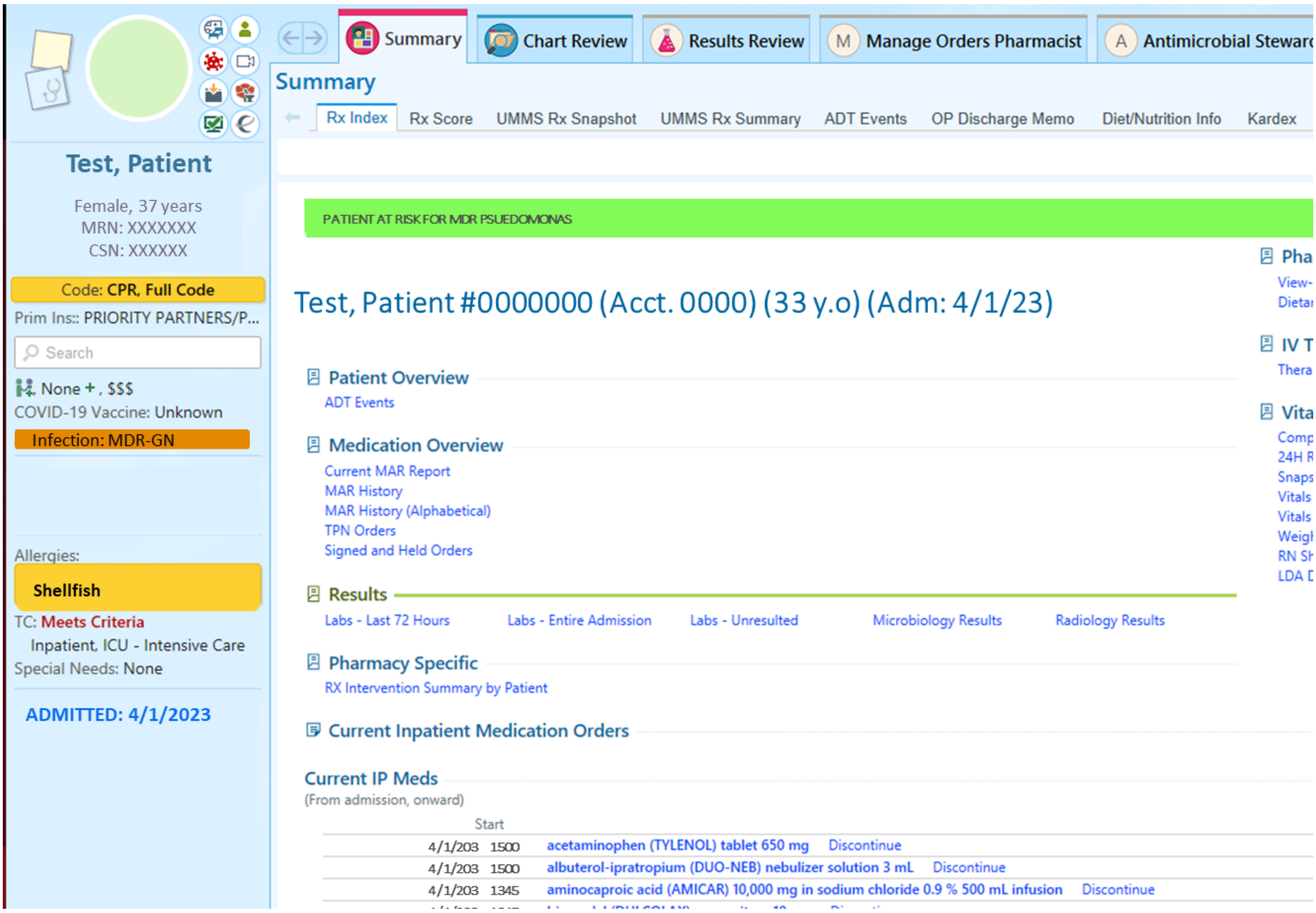

Would the reported information be adequate for clinical decisions regarding escalation of therapy for MDR P. aeruginosa?

- ◯ Strongly disagree
- ◯ Disagree
- ◯ Neither agree nor disagree
- ◯ Agree
- ◯ Strongly agree

Are there aspects of the tool that should or should not be altered to make it more effective? What do you find useful or not useful?

____

BestPractice Advisory - Test, Flannagin Ummc-B

Informational (1)

Your patient recently had a clinical culture with *P. aeruginosa* identified and is at risk for multi-drug resistance because of:

1. Previous MDR *P. aeruginosa* in the last 6 months
2. Multiple course of anti-pseudomonal antibiotics
3. Infection presenting after multiple days of hospitalization

Consider escalation of antibiotic therapy to cover mutli-drug resistance P. aeruginosa

✔ Accept | Dismiss

Would the reported information be adequate for clinical decisions regarding escalation of therapy for MDR P. aeruginosa?

- ◯ Strongly disagree
- ◯ Disagree
- ◯ Neither agree nor disagree
- ◯ Agree
- ◯ Strongly agree

Are there aspects of the tool that should or should not be altered to make it more effective? What do you find useful or not useful?

____

Results

Bronchial Culture w Gram Stain

Status: Preliminary result
Specimen information: Bronchoalveolar Lavage
Order: 212475539

Component

PRE

!

```
>10,000 CFU/mL Pseudomonas aeruginosa
Patient is at risk for multi-drug resistant Pseudomonas aeruginosa. Consider
ID consult
```

Gram Stain

```
Abundant polymorphonuclear leukocytes
No epithelial cells seen
Gram-negative rods
```

Specimen Collected: XX/XX/2023

Would the reported information be adequate for clinical decisions regarding escalation of therapy for MDR P. aeruginosa?	◯ Strongly disagree<br>◯ Disagree<br>◯ Neither agree nor disagree<br>◯ Agree<br>◯ Strongly agree
Are there aspects of the tool that should or should not be altered to make it more effective? What do you find useful or not useful?	__________________________________
What kinds of infrastructure changes will be needed to accommodate implementation of these new criteria and tools?	☐ Changes in scope of practice to allow executing changes in empiric prescribing.<br>☐ Formal policies to change empiric prescribing patterns.<br>☐ Clinical detailing to educate on risk for MDR P. aeruginosa.<br>☐ Clinical detailing to educate on changes in the EMR.<br>☐ Other
Describe if 'other'	____________________________
What is your clinical role?	◯ Clinical pharmacist<br>◯ Fellow<br>◯ ICU physician<br>◯ Mid-level provider<br>◯ Resident
How many years have you been in practice?	◯ 1-3 years<br>◯ 3-5 years<br>◯ 5-10 years<br>◯ >10 years
Where is your primary place of work?<br><br>Choose up to two	☐ Medical ICU<br>☐ Neuro ICU<br>☐ Surgical ICU<br>☐ Trauma ICU<br>☐ Cardiac surgery ICU<br>☐ Cardiac care unit
Thank you for completing the survey!<br><br>If you would like to receive a $20 e-gift card, please enter your email address	____________________________
