# Supplementary material for: Crossroads of Antimicrobial and Diagnostic Stewardship: Assessing Risks to Develop Clinical Decision Support to Combat Multidrug-Resistant Pseudomonas
Source: Open Forum Infect Dis. 2023 Oct 12;10(10):ofad512. doi: 10.1093/ofid/ofad512 (PMC10603593; doi:10.1093/ofid/ofad512)
Supplement: ofad512_Supplementary_Data [file ofad512_supplementary_data.zip › MDRP Supplementary Appendix.docx]

**Supplementary Appendix: Data Dictionary**

- **MDRP Definition:** *Pseudomonas aeruginosa* non-susceptible to 2 of the following 3: Cefepime, Imipenem or Meropenem or Doripenem or Ertapenem, Piperacillin/Tazobactam
- **Age:** Age at time of *P. aeruginosa* culture, years (continuous) AND category: 18 – 25/26 – 35/ 36 – 45/ 46
- **ICU**: present in ICU (based on documented patient location) at time of culture collection or admitted to ICU between culture collection and organism identification/resulting in EMR
- **Infection Site:** Based on culture collection documentation in EMR, category: HBAP/VAP (sputum, BAL, tracheal aspirate), versus BSI (blood)
- **POA Infection:** Culture collected within 72 hours of admission
- **Transfer from OSF: Documentation of transfer to acute care hospital, category: OSH, SNF, Other**
- **Previous hospital admission:** Documented in EMR within 6 months of index visit, not including index visit
- **Previous ICU admission:** Documented in EMR within 6 months of index visit, not including index visit
- **Previous MDR GN:** Any multi-drug resistant gram-negative (ESBL/AmpC, CRE, MDRP, carbapenem-resistant A. baumannii) isolated from clinical or surveillance cultures in the six months prior to index visit, not including index visit, category: surveillance (swabs) versus clinical isolate.
- **Prior *P. aeruginosa* infection:** Prior clinical culture with isolation of *P. aeruginosa* with documentation of clinically treated infection, regardless of source, in the previous 3 months prior to index visit
  - Within 30 days of culture with isolation of *P. aeruginosa?*
  - History carbapenem resistant *P aeruginosa* infection within 30 days?
- **Endotracheal tube:** Documented presence of endotracheal tube prior to collection of culture growing *P. aeruginosa*
- **Tracheostomy:** Documented presence of tracheostomy tube prior to collection of culture growing *P. aeruginosa*
- **Hemodialysis:** Receipt of renal replacement therapy either inpatient or outpatient prior to collection of culture growing *P. aeruginosa*
- **PICC, CVC or port:** Documented insertion or presence of peripherally inserted central catheter, central venous catheter, or surgically implanted port catheter prior to collection of culture growing *P. aeruginosa*
- **Previous antibiotics:** Receipt of at least one dose of systemic antibiotics in the 30 calendar days prior, but not within 24 hours of prior to collection of culture growing *P. aeruginosa* (includes documentation in medication administration records or past medical history per EMR)
  - Collection of receipt of at least one dose of systemic:
    - Fluoroquinolones (ciprofloxacin, levofloxacin, ofloxacin)
    - Anti-pseudomonal cephalosporin (cefepime, ceftazidime, ceftazidime/avibactam, ceftolozane/tazobactam)
    - Anti-pseudomonal carbapenem (meropenem, imipenem/cilastatin, doripenem)
    - Piperacillin/tazobactam
  - Documentation of how many classes of antibiotics with Gram-negative activity they had been exposed to in the prior 30 days, category, 0 to 1, 2 to 3, 4 or more
- **Comorbid conditions:** Present at time of collection of culture growing *P. aeruginosa,* as documented in patient past medical history per EMR and aligned with definitions from Lodise et al., category (yes/no)
  - Diabetes with complications (documented retinopathy, nephropathy, neuropathy, macrovascular complications secondary to diabetes)
  - Diabetes without complications
  - Peripheral vascular disease
  - Paraplegia and hemiplegia
  - Chronic pulmonary disease
  - Myocardial infarction
  - Cancer
  - Cerebrovascular disease
  - Congestive heart failure
  - Mild liver disease
